# Serum protein profiles and C-reactive protein in natural canine filariasis

**DOI:** 10.14202/vetworld.2021.860-864

**Published:** 2021-04-10

**Authors:** Sariya Asawakarn, Sujin Sirisawadi, Nanthida Kunnasut, Patchana Kamkong, Piyanan Taweethavonsawat

**Affiliations:** 1 Biochemistry Unit, Department of Veterinary Physiology, Faculty of Veterinary Science, Chulalongkorn University, Bangkok, 10330, Thailand; 2 Parasitology Unit, Department of Veterinary Pathology, Faculty of Veterinary Science, Chulalongkorn University, Bangkok, 10330, Thailand

**Keywords:** *Brugia pahangi*, C-reactive protein, *Dirofilaria immitis*, dogs, serum protein

## Abstract

**Background and Aim:**

Canine filariasis is caused by several species of filarial worms. The pathophysiological response to infection is mainly due to the filaria lifecycle. Laboratory detection methods to assess the pathological alterations characteristic of filariasis are needed urgently. Serum protein profiles and C-reactive protein (CRP) levels are used widely to diagnose several animal diseases. This study aimed to determine the serum protein profiles and CRP levels in dogs infected with *Dirofilaria immitis* or *Brugia pahangi* or both parasites.

**Materials and Methods:**

Blood samples were collected from 980 dogs presenting at animal hospitals and veterinary clinics in Bangkok and its vicinity. The presence of microfilaria in samples was determined using a buffy coat smear and staining with Wright–Giemsa. The sheathed and unsheathed microfilaria species were identified by acid phosphatase staining. Forty positive samples were tested. The serum protein profiles were identified by agarose gel electrophoresis. The CRP concentration was measured using a fluorescent immunoassay.

**Results:**

Albumin levels and albumin-to-globulin ratios were significantly lower, and total protein, β2 globulin, and γ globulin levels were significantly elevated in dogs infected with *D. immitis* and *B. pahangi* compared with reference values in normal dogs. The average CRP concentrations in dogs infected with *D. immitis* or *B. pahangi* were 69.9 and 12.9 mg/L, respectively.

**Conclusion:**

The total protein and γ globulin levels increased in canine filariasis compared with the normal reference range. The CRP concentration in dogs infected with *D. immitis* was extremely high, whereas that in dog infected with *B. pahangi* was normal.

## Introduction

Canine filariasis is a prominent mosquito-borne disease that occurs worldwide, including in Thailand. Filariasis is a considerable public health concern in tropical and subtropical areas. Several species of filarial worms, including *Dirofilaria immitis*, *Dirofilaria repens*, *Brugia pahangi*, and *Acanthocheilonema reconditum*, can cause filariasis. These species have been reported in Thailand [1]. *D. immitis* infection, which causes canine heartworm disease, is the most pathogenic filarial parasite. The clinical signs of filariasis are exercise intolerance, coughing, ascites, and heart failure. The pathophysiological response to heartworm infection is mainly due to the living adult worms in the pulmonary arteries and right ventricle. In addition, dead microfilaria and released adult worms can release an endosymbiotic bacterium, named *Wolbachia pipientis*. This bacterium is crucial to the pathophysiological response to canine heartworm disease. The severity of infection is characterized by endocarditis, with intimal proliferation and thickened vessel walls [2,3]. *Brugia* spp. cause lymphatic filariasis; in humans, these pathogens, especially *Brugia malayi*, cause elephantiasis. In pets, *B. pahangi* is transmitted by mosquitoes and its lifecycle is similar to that of other filarial worms. Infective larvae enter peripheral lymphatics, migrate to the nearest lymph node, and develop for 2 weeks before migrating to other lymphatics, where the larvae mature and produce lymphadenitis, granulomatous lymphangitis, and lymphangiectasia. Notably, in dogs, *B. pahangi* does not cause elephantiasis as it does in humans. Infected dogs are generally asymptomatic, although lymphadenopathy and lymphedema have been reported in some cases [4,5].

The development of laboratory methods to assess the pathological alterations characteristic of the disease is needed urgently. In addition, new tools are needed to determine the health status of dogs infected with *D. immitis* or *B. pahangi*, including tools for disease staging and accurate prognoses. Serum protein profiles and inflammatory biomarkers are suitable tools to monitor infected animals. Serum protein profiles are useful for veterinarians to monitor the health status of animals. Major canine serum proteins are separated into five or six bands, including albumin, α1 globulin, α2 globulin, β globulin, and γ globulin. The β globulin fraction can separate into the β1 globulin and β2 globulin fractions. Serum protein profiles have been used widely as a diagnostic tool to monitor the status of infectious and other diseases, such as multiple myeloma in dogs [6-10].

The acute-phase response is an early defense of the body in response to trauma, inflammation, or infection. The acute-phase response is part of the innate host defense system and systemic effects, including fever, leukocytosis, and increased blood cortisol. C-reactive protein (CRP) is one of the acute-phase proteins. CRP was first described as an acute-phase protein in 1930 and named for its ability to bind to C-polysaccharide from *Pneumococcus pneumonia* [11,12]. Pro-inflammatory cytokines, such as interleukin 6 (IL-6) and tumor necrosis factor-alpha (TNF-a), stimulate the liver to produce CRP and release it into the bloodstream. CRP is a major acute-phase protein in dogs; however, information on serum protein profiles and CRP levels in dogs infected with *D. immitis* (i.e., canine heartworm disease) or *B. pahangi* is limited.

The aim of this study was to determine and to compare the serum protein profiles and CRP level in dogs infected with *D. immitis* or *B. pahangi* with normal reference values from healthy dogs, with the goal of utilizing these parameters to monitor disease status.

## Materials and Methods

### Ethical approval

The research protocol was approved by Chulalongkorn University Animal Committee (approval no. 1931052).

### Study period and location

Samples were collected from private Small Animal Hospitals and Veterinary Clinic in Bangkok from August 2019 to July 2020.

### Sample collection

Blood samples were collected from 980 canines (*Canis familiaris*). Some dogs exhibited the clinical signs of filariasis, including exercise intolerance, coughing, and ascites. However, most dogs did not exhibit any clinical signs of filariasis. All samples were tested to determine the presence of microfilaria using buffy coat blood smears and staining with Wright–Giemsa. Sheathed and unsheathed microfilaria were identified to determine the filarial species using acid phosphatase staining [1]. The number of dogs positive for *D. immitis* or *B. pahangi* or both parasites was 24, 15, and 1, respectively. All canine blood samples were collected in serum collection tubes. All serum samples were kept at –20ºC until analyses.

### Determination of the serum protein profile by electrophoresis

Total protein concentrations in the 40 positive serum samples were determined using a photometric colorimetric test or a biuret method test kit (Human^®^, Wiesbaden, Germany). Proteins were separated by agarose gel electrophoresis (SPIFE^®^ Split Beta SPE kit, Helena Laboratories, Beaumont, Texas, USA) to examine the serum protein profiles. 1.3 milligram protein of each serum sample were electrophoresed at 400 V for 6 min. The gels were pre-dried at 53°C for 12 min, stained with acid blue staining solution, and destained with citric acid using an automated machine (Spife^®^ 3000, Helena Laboratories). The density of protein bands was analyzed using the Quickscan Touch program (Helena Laboratories). The specimen electrophoresis protein serum (SPEP) data from dogs infected with *D. immitis* or *B. pahangi* were compared to the reference ranges of normal, uninfected dogs reported by Kaneko [13].

### Measurement of CRP concentration

The CRP concentrations were measured using a fluorescent immunoassay (Vcheck Canine CRP 2.0 Test Kit, Bionote, Gyeonggi-do, South Korea). The CRP concentrations were determined in serum samples from dogs infected with *D. immitis* (n=6) or *B. pahangi* (n=6) or both parasites (n=1). Five microliters of each sample were diluted with the diluent buffer (to 100 μL), mixed, and added to the test device (V200 Analyzer, Bionote, South Korea). A CRP concentration of more than 30 mg/L represented an abnormal value.

### Statistical analysis

SPEP data from infected dogs were analyzed using a general linear model (GLM) with the online SAS version 9.4 (SAS Inst. Inc., Cary, NC, USA) or SAS University Edition (https://www.sas.com/en_us/software/university-edition.html). The GLM was y=trt+method+trt*method+e, where *y* was the serum protein value from each filarial worm species and method of use, *trt* is a factor from each filarial worm species, *method* is a factor from the laboratory technique used to measure serum protein profiles, trt*method is the interaction term between the two factors, and *e* is the residual value from each observation.

## Results

**Figure-1:**
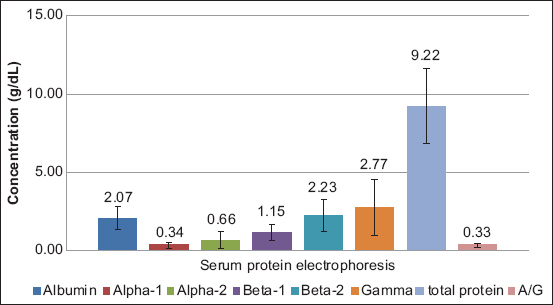
The albumin, α1 globulin, α2 globulin, β1 globulin, β2 globulin, γ globulin, and total protein concentrations (mean±standard deviation in g/dL) and albumin-globulin (A/G) ratio of dogs infected with *Dirofilaria immitis*. The numbers indicate the average values.

**Figure-2:**
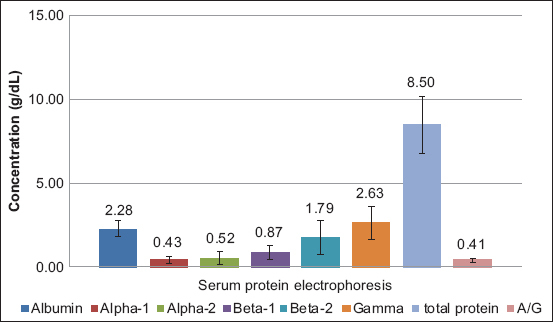
The albumin, α1 globulin, α2 globulin, β1 globulin, β2 globulin, γ globulin, and total protein concentrations (mean±standard deviation in g/dL) and albumin-globulin (A/G) ratio of dogs infected with *Brugia pahangi*. The numbers indicate the average values.

**Figure-3:**
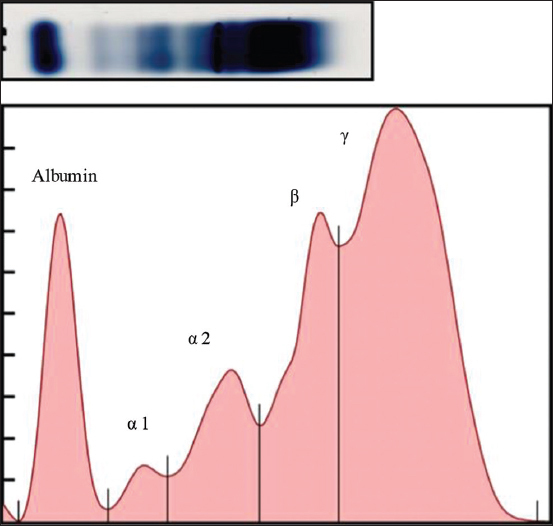
A representative electrophoretogram (top) of an infected with *Dirofilaria immitis*. The serum protein profile (bottom) shows the reduced albumin peak, increased β globulin, and γ globulin peaks (hyperglobulinemia).

**Figure-4:**
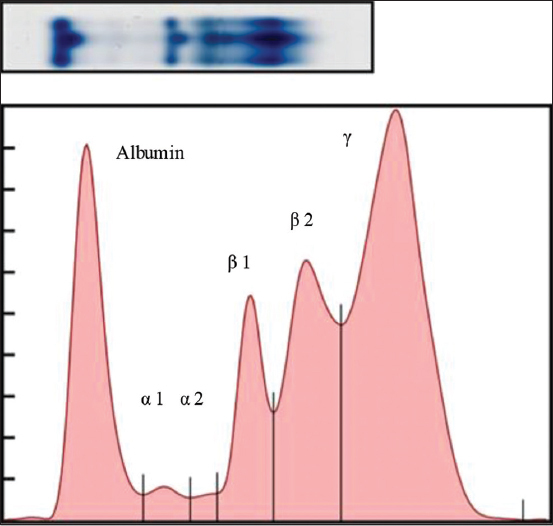
A representative electrophoretogram (top) of a dog infected with *Brugia pahangi*. The serum protein profile (bottom) shows the elevated β globulin and γ globulin peaks.

**Figure-5:**
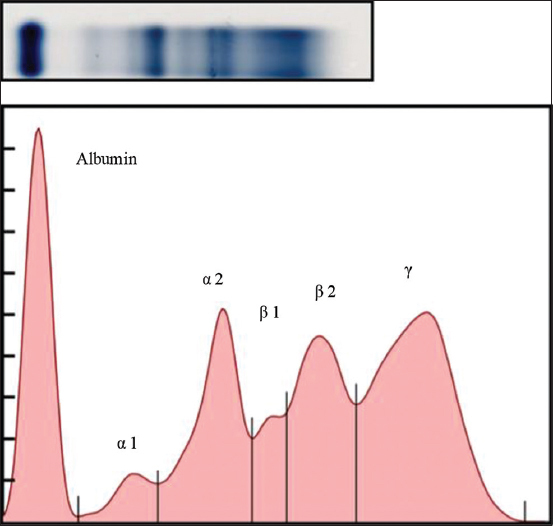
A representative electrophoretogram (top) of a dog infected with *Dirofilaria immitis* and *Brugia pahangi*.

Dogs infected with *D. immitis* and *B. pahangi* showed significantly lower albumin levels and albumin-to-globulin (A/G) ratios and significantly ­elevated total protein, β2 globulin, and γ globulin levels compared with reference values of normal dogs (Table-1). The serum protein electropherograms are shown in Figures-1-4. The serum protein electrophoretogram for dogs infected with both is shown in Figure-5.

**Table-1:** The total protein, albumin, α1 globulin, α2 globulin, β1 globulin, β2 globulin, and γ globulin levels and albumin-globulin (A/G) ratio.

Variable (g/dL)	*Dirofilaria immitis* positive (mean±SD) (n=24)	*Brugia pahangi* positive (mean±SD) (n=15)	Reference range [13]
Total protein	9.22±2.40	8.50±1.75	5.40-7.10
Albumin	2.07±0.70	2.28±0.50	2.60-3.30
Alpha-1	0.34±0.17	0.43±0.18	0.20-0.50
Alpha-2	0.66±0.55	0.52±0.45	0.30-1.10
Beta-1	1.15±0.53	0.87±0.46	0.70-1.30
Beta-2	2.23±1.02	1.79±0.06	0.60-1.40
Gamma	2.77±1.84	2.63±1.17	0.90-2.20
A/G ratio	0.33±0.12	0.41±0.15	0.59-1.11

The CRP concentrations of dogs infected with only *D. immitis* (n=6) were between 13.6 and 116.9 mg/L, with an average of 69.6 mg/L. In addition, dogs had high CRP concentration; they may have to monitor health status during the treatment process. There were four samples with CRP concentrations above 30 mg/L and two samples below 30 mg/L. The CRP concentrations of dogs infected with *B. pahangi* (n=6) were in the normal range (< 30 mg/L): <10 mg/L (n=4), 18.8 mg/L (n=1), and 31 mg/L (n=1). The average CRP concentration was 12.9 mg/L in dogs infected with *B. pahangi*. The CRP concentration in the dog infected with both *D. immitis* and *B. pahangi* was >200 mg/L.

## Discussion

Determining the serum protein profile is a basic test used in animal hematology and clinical chemistry to monitor the patient’s health and disease status. Variations in serum protein profiles commonly occur as secondary symptoms in numerous diseases and may be the primary symptom of certain conditions. Several factors may influence the concentration of serum proteins. Serum protein profiles are an excellent method to detect acute or chronic inflammation and stimulate humoral immune response [6,14].

The Spilt Beta SPE Kit separates serum proteins into five or six bands: Albumin, α1 globulin, α2 globulin, β globulin, and γ globulin. The β globulin peak can also be split into β1 and β2 globulin peaks. Each globulin fraction consists of acute-phase proteins or antibodies, and sometimes both [15]. In dogs infected with *D. immitis* or *B. pahangi*, albumin levels and the A/G ratio were reduced compared with normal reference dog values [13]. Reduced albumin levels are usually caused by starvation, liver insufficiency, kidney disease, congestive heart failure, or parasitic disease [6,8,10]. Canine filariasis leads to marked changes in the parameters used to evaluate liver and kidney functions [16]. A reduced A/G ratio is caused by the overproduction of globulins. In this study, total protein and γ globulin levels were elevated in dogs infected with *D. immitis* or *B. pahangi* compared with the levels in healthy dogs [13]. The high level of total proteins is indicative of hyperglobulinemia because of the humoral response induced by *D. immitis*. The high level of γ globulin indicates the high production of immunoglobulins (Igs), such as IgG, in response to chronic inflammation. Hyperglobulinemia can be caused by chronic infection, inflammation, neoplasia, and parasitic diseases, such as dirofilariasis, scabies, and *Ehrlichia* infection [17]. Clinical signs of heartworm disease appear as chronic infection and marked allergic response to the adult worms and microfilariae. Hyperglobulinemia is an expected feature of canine heartworm disease and can be either polyclonal or monoclonal gammopathy. We noted clonal gammopathy using the SPE kit, which identified the monoclonal protein IgG. Dirofilariasis has been hypothesized to elicit the atypical benign clonal proliferation of plasma cells [18]. In dogs, γ globulin fractions are composed of various classes of Igs, and CRP also migrates in this fraction [19,20]. Moreover, the elevated γ globulin level in dogs infected with only *D. immitis* might be related to the high CRP concentration. Similar to dogs infected with *D. immitis*, dogs infected with only *B. pahangi* showed a reduced albumin level and A/G ratio, and elevated total protein, β2 globulin, and γ globulin levels compared with reference levels in healthy dogs [13]. In humans infected with *Loa loa*, total serum protein β globulin and γ globulin fractions with monoclonal gammopathy are elevated. The albumin, A/G ratio, and α-globulin were all normal in dogs with *B. pahangi* [21].

CRP is a major canine acute-phase protein that increases rapidly in a wide range of inflammatory conditions; CRP is especially high in chronic conditions. From our results, the average CRP concentration in dogs infected with only *D. immitis* was higher than the reference range, but the CRP concentration was normal in dogs infected with only *B. pahangi*. The CRP concentration in dogs infected with both *D. immitis* and *B. pahangi* was markedly elevated (> 200 mg/L). A striking increase in CRP and decrease in albumin and paraoxonase-1 activity have been observed in canine heartworm disease [22]. CRP levels are elevated in canine heartworm disease because of inflammation involved in the pathogenesis [23]. Heartworm disease leads to pulmonary hypertension and, in the late stage, may induce right-side cardiac insufficiency. Adult worms are localized in the pulmonary arteries, which induces endothelial damage or proliferative endocarditis. The CRP concentration can be used as a marker of endothelial arteritis and pulmonary hypertension in dogs infected with *D. immitis* [24]. However, the CRP concentration in dogs infected with *B. pahangi* was in the normal range; those dogs were mostly asymptomatic. In humans, the CRP concentration in patients with asymptomatic microfilaremia was lower than in patients with chronic lymphatic pathology. The patients with chronic lymphatic obstruction caused by the filarial parasite *Wuchereria bancrofti* had elevated serum CRP [25]. The extremely high CRP concentration in the coinfected dogs indicates that the inflammatory response may be caused by *D. immitis* infection.

## Conclusion

The serum protein profiles and CRP concentrations in canine filariasis can reflect the health status of infected dogs. The total protein and γ globulin levels increased in canine filariasis compared with the normal reference range. The CRP concentrations in dogs infected with *D. immitis* were extremely high, whereas those in dogs infected with *B. pahangi* were normal. This information can be utilized by veterinarians to monitor infected dogs during treatment.

## Authors’ Contributions

SA and PT designed the experiments. NK, SS, and PK contributed to the analysis and interpretation of data. SA and PT wrote the manuscript. NK, SS, and PK assisted in writing and revision of the manuscript. All authors have read and approved the final manuscript.
